# Saberes previos de los estudiantes: aspectos esenciales que un docente experto en enfermería identifica, interpreta y organiza para favorecer el aprendizaje

**DOI:** 10.17533/udea.iee.v42n2e02

**Published:** 2024-07-03

**Authors:** Marcela Carrillo Pineda, Alexandra María Bolívar Zapata, José Luis Medina Moya, Margarita María Gómez Gómez, Águeda Lucía Valencia Deossa, Teresita Alzate-Yepes

**Affiliations:** 1 Nurse, Ph.D. Professor, Faculty of Nursing. Email: marcela.carrillo@udea.edu.co. Universidad de Antioquia Faculty of Nursing Colombia marcela.carrillo@udea.edu.co; 2 Nurse, Master’s degree. Professor, Faculty of Nursing. Email: alexandra.bolivar@udea.edu.co Universidad de Antioquia Faculty of Nursing Colombia alexandra.bolivar@udea.edu.co; 3 BE in Pedagogy and Nurse, Ph.D. Full Professor, Faculty of Education, Universidad de Barcelona (Spain). Email: jlmedina@ub.edu Universitat de Barcelona Faculty of Education Universidad de Barcelona Spain jlmedina@ub.edu; 4 Social Communicator and BE in Education, Master’s degree. Professor, Faculty of Nursing. Email: margaritam.gomez@udea.edu.co Universidad de Antioquia Faculty of Nursing Colombia margaritam.gomez@udea.edu.co; 5 Nurse, Master’s degree. Professor, Faculty of Nursing. Email: agueda.valencia@udea.edu.co Universidad de Antioquia Faculty of Nursing Colombia agueda.valencia@udea.edu.co; 6 Nutritionist and Dietitian, PhD. Professor, School of Dietetics and Nutrition. Email: teresita.alzate@gmail.com Universidad de Antioquia School of Dietetics and Nutrition Colombia teresita.alzate@gmail.com; 7 Universidad de Antioquia, Medellín, Colombia. Universidad de Antioquia Universidad de Antioquia Medellín Colombia

**Keywords:** nursing education, nursing student, learning, nursing faculty., educación en enfermería, estudiantes de enfermería, aprendizaje, docentes de enfermería., educação em enfermagem, estudantes de enfermagem, aprendizagem, docentes de enfermagem.

## Abstract

**Objetivo.:**

Analizar los aspectos esenciales que el docente experto en enfermería identifica, interpreta y organiza durante los procesos dialógicos con los estudiantes en el aula para favorecer su aprendizaje.

**Métodos.:**

Estudio cualitativo, parte de un estudio multicéntrico, que utilizó la etnografía de la comunicación desde un abordaje micro-etnográfico. Se seleccionó un docente experto de una Facultad de Enfermería de una universidad pública de Medellín, Colombia. El trabajo de campo se desarrolló en tres momentos: 1) observaciones no participantes en dos clases presenciales del curso de morfofisiología grabadas en video en dos planos (uno enfocando al profesor, y el otro, a los estudiantes); 2) entrevistas Think-Aloud Protocol (método de pensamiento en voz alta) al docente y a cinco estudiantes (tres de la primera clase y dos de la segunda) que iniciaron espontáneamente más de dos interacciones con el docente durante las clases; y 3) transcripciones paralelas, organizadas en secuencias didácticas (videos). El análisis se apoyó en la unidad [E-P(i-e-r)E´] (Estudiante-Profesor (identificación-evaluación-respuesta) Estudiante´) y en comparaciones constantes de los datos.

**Resultados.:**

Emergieron cuatro categorías: 1) Identificación de aspectos esenciales: importancia de los saberes previos, 2) Interpretación: articulación de los aspectos esenciales y los procesos mentales del estudiantado, 3) Organización de la respuesta: conexión entre saberes previos y el nuevo conocimiento, y: 4) Sintonización con las necesidades de aprendizaje del estudiantado; las que fueron agrupadas en una meta-categoría: Saberes previos del estudiante: aspectos esenciales para el aprendizaje.

**Conclusión.:**

Los saberes previos experienciales de los estudiantes se constituyen en los aspectos esenciales identificados, interpretados y organizados por el profesor experto, para el logro de aprendizajes significativos.

## Abstract (en)

**Objective.:**

To analyze the essential aspects that the nursing expert professor identifies, interprets, and organizes during classroom dialogic processes with students to foster their learning.

**Methods.:**

Qualitative study, part of a multicenter study, which used ethnography of communication specifically from a micro-ethnographic approach. An expert professor from the Faculty of Nursing at a public university in Medellín, Colombia, was selected for the study. The fieldwork was done in three stages: 1)non-participant observations in two in-person classes of the Morphophysiology course recorded on video from two different perspectives (one focusing on the professor and another on the students); 2)think-aloud interviews with the professor and five students (three from the first class and two from the second) who spontaneously started more than two communicative interactions with the professor during the classes; and 3)parallel transcriptions, organized in didactic sequences (videos). The analysis was supported by the unit Student-Professor (identification-evaluation-answer) Student [S-P(i-e-a)S´], and by continuous comparisons of the data.

**Results.:**

Four categories were identified: 1) Identification of essential aspects: importance of prior knowledge, 2) Interpretation: connection between essential aspects and students’ mental processes, 3) Organization of the answer: connection between prior knowledge and new knowledge, and: 4) Synchronization with the learning needs of the students, which were grouped in a meta-category: Prior knowledge of the students: essential aspects for learning.

**Conclusion.:**

Students’ experiential prior knowledge constitutes the essential aspects identified, interpreted, and organized by the expert professor to achieve significant learning.

## Resumo

**Objetivo.:**

Analisar os aspectos essenciais que o professor especialista em enfermagem identifica, interpreta e organiza durante os processos dialógicos com os alunos em sala de aula para promover sua aprendizagem.

**Métodos.:**

Estudo qualitativo, parte de um estudo multicêntrico, que utilizou a etnografia da comunicação a partir de uma abordagem microetnográfica. Foi selecionada uma professora especialista da Faculdade de Enfermagem de uma universidade pública de Medellín, Colômbia. O trabalho de campo foi desenvolvido em três momentos: 1) observações não participantes em duas aulas presenciais do curso de morfofisiologia gravadas em vídeo em dois planos (um focado no professor, e outro, nos alunos); 2) Entrevistas do Protocolo Think-Aloud com a professora e cinco alunos (três da primeira turma e dois da segunda) que iniciaram espontaneamente mais de duas interações com a professora durante as aulas; e 3) transcrições paralelas, organizadas em sequências didáticas (vídeos). A análise baseou-se na unidade [E-P(i-e-r)E´] (Aluno-Professor (identificação-avaliação-resposta) Aluno´) e em comparações constantes dos dados.

**Resultados.:**

Emergiram quatro categorias: 1) Identificação dos aspectos essenciais: importância do conhecimento prévio, 2) Interpretação: articulação dos aspectos essenciais e dos processos mentais do aluno, 3) Organização da resposta: conexão entre conhecimentos prévios e novos conhecimentos, e: 4) Sintonia com as necessidades de aprendizagem do corpo discente; que foram agrupados em uma metacategoria: Conhecimentos prévios do aluno: aspectos essenciais para a aprendizagem.

**Conclusão.:**

. O conhecimento experiencial prévio dos alunos constitui os aspectos essenciais identificados, interpretados e organizados pelo professor especialista, para a obtenção de uma aprendizagem significativa.

## Introduction

The development of teaching expertise is a central topic of current discussion in nursing education. It has been shown that, for nursing teaching practice, clinical experience^1^ or postgraduate training in education (masters or doctoral degrees) are not enough by themselves,^1^^,^^2^ since general didactics does not cover the specificities necessary to teach the concepts inherent to each discipline.^2^The role of the nursing professor implies not only having professional practice competence, which is what provides knowledge on the subject, but also developing knowledge and skills regarding the specific ways nursing is taught, learned, and evaluated;^1^ since, as any other discipline, it requires specific knowledge that responds to its own pedagogical needs. This is the Pedagogical Content Knowledge (PCK), which, according to Shulman^3^ represents “the blending of content and pedagogy into an understanding of how particular topics, problems, or issues are organized, represented, and adapted to the diverse interests and abilities of learners.” PCK analysis allows establishing the relationship that exists between scientific, pedagogical and didactic knowledge in the teaching of any discipline,^4^ and it is easily identifiable in an expert professor,^3^ who stands out for having extensive knowledge about the topics, about the strategies for teaching them, and about the students and their prior knowledge^4^, to transform them into pedagogical representations and actions.^3^

In recent years, PCK has been studied in education sciences^4^^-^^6^ and in higher education,^2^^,^^7^^-^^10^ to understand the construction of expert teaching knowledge^4^^,^^6^^-^^8^ and the most appropriate PCK models in specific fields.^2^^,^^4^^,^^5^^,^^7^ Although some of these studies^6^^,^^9^^,^^10^ have considered pedagogical practice in relation to learners, more empirical evidence is still needed, particularly in nursing, in order to understand the aspects of PCK that contribute to learning in the context of the pedagogical interaction between professors and learners.^10^ Given the above and recognizing the intellectual, relational, and emotional nature of PCK,^11^ the need to deepen the knowledge in real time of the dialogic-reflexive processes, between the disciplinary and pedagogical knowledge of the professor in nursing education and between these and the student’s learning, was identified. Dialogic processes, in this case, refer to those moments when the learner starts the communicative process with a question and the professor answers, thus opening a dialogue on the topic during the class. 

Accordingly, the objective of this study was to analyze the essential aspects that the nursing expert professor identifies, interprets, and organizes during the dialogic process with the students in the classroom to foster their learning. Analyzing the essential aspects of nursing learning in PCK, in the context of the different linguistic approaches of the expert professor and the students and how they are connected, allows delving into the didactic principles that take place in that specific learning setting.^2^ These principles could support the content relevant to nursing teaching^1^ and the identification of the elements involved in learning its object of study^4^ as situated, contextualized, meaningful knowledge relevant to the learning students’ care needs.

## Methods

A qualitative research study using communication ethnography with a micro-ethnographic approach based on micro-sociolinguistics^12^ was conducted as part of a multicenter study entitled “Disciplinary knowledge, pedagogical knowledge and situated learning: origin and mutual influences in university teaching” [*Saberes disciplinares, saberes pedagógicos y aprendizaje situado: génesis e influencias mutuas en la enseñanza universitaria*]. In this study, different universities from Spain, Chile, Brazil, and Colombia (Medellín and Bogotá) collaborated. The principal investigator of the multicenter study, who led and supported the entire project, was a university professor, a nurse, BE in Education and PhD in Philosophy and Education Sciences. Approximately eight meetings (both online and in-person) were held with the research team at the beginning of the project for training and methodology standardization.

This paper presents the results of a research study carried out between 2018 and 2019 at the Faculty of Nursing of a public university in Medellín, Colombia. The multidisciplinary team in charge of collecting and analyzing the data at all stages of the project consisted of three nurses, two with a master’s degree and one with a PhD; a social communicator with a master’s degree; and a dietitian nutritionist with a PhD. All the researchers were university professors trained in qualitative research and with experience in research studies in education. 

The expert professor, the subject of the research, was selected in the Faculty of Nursing by means of three mechanisms: (i) *Individual anonymous face-to-face survey* given in class to 63 students in the last year of the undergraduate nursing program, in which they were asked to identify, with arguments, the three best professors during their learning process who, in addition, had used participatory strategies in the classroom. Students identified the 43 best professors. (ii) *Individual anonymous Google form survey* given to 24 professors from the Faculty of Nursing, in which they were asked to name three colleagues known as the best professors and argue their selection. The professors identified 28 colleagues. (iii) Prioritization of the 13 best professors identified by students and professors and selection of the Morphophysiology course professor, who was named the most, 31 times. The academic community chose this professor because of his broad knowledge of nursing practice, his ability to teach students by means of playful and creative didactic resources, in an environment of calm and trust, and because of his encouragement of students’ interest, motivation, and learning. The selection criteria for the students who participated in the fieldwork were to be a student of the Faculty of Nursing and to be enrolled in the Morphophysiology course taught by the selected professor. 

The fieldwork was done in three stages: 

### First stage

To identify the interactions between the professor and the students, the researchers made non-participant observations in two in-person classes of the Morphophysiology course taught by the expert professor on the topic of cardiology to two groups of almost 30 students. Each observation lasted approximately two hours, and both were recorded by an audiovisual specialist from two different perspectives: one focusing on the professor and another one on the students. The observation of each class was carried out by three researchers located in different parts of the classroom seeking to identify the students who had more verbal interactions with the professor starting with a question; these students’ data were collected to contact them later for an interview.

Using the class recordings from two perspectives, contrasted on the same screen, the moments in which the 28 interactions between professors and students took place were identified. These interactions were named *episodes* with the sequence Student-Professor (S-P) because they were spontaneously initiated by the student. Each episode was organized into a three-part video: classroom context prior to the S-P interaction, student question, and answer from the professor. Of the 28 interactions, 23 episodes with S-P interaction were selected (13 from the first class and 10 from the second), because those in which the same student starred in more than two interactions were prioritized, and the 5 episodes in which the student only had one S-P sequence were discarded.

### Second stage

Think-aloud interviews^13^ were conducted with the professor and the five students (three from the first class and two from the second) who starred in the 23 selected episodes, in which they verbally expressed what they were thinking during the S-P interaction while they were watching the episode in which they were protagonists.^12^The interviews were conducted in a classroom within the institution and were also videotaped. In some of the interviews two undergraduate students and one graduate student linked to the study were present as research trainees, as well as the audiovisual assistant who recorded and edited the videos.

Of the 23 episodes, fourteen think-aloud interviews were conducted with the professor subject of the study, since the desired saturation level was achieved. The interviews averaged 25 minutes and were done in two sessions of three hours each. They were conducted two days after each class. While the researchers showed the videos to the professor, they asked him questions such as: What was the first thing you thought when you heard the student’s question? Did you understand the student’s question? What elements of the question did you pay the most attention to? What criteria did you use to organize the ideas and elaborate the answer? And what did you intend with the answer?

Regarding the students, fourteen think-aloud interviews were also conducted (7 from each class) with the same episodes used with the professor. Student interviews lasted an average of 15 minutes and were conducted three days after each class. In this case, while the researchers showed them the videos, they answered questions such as: What did you want to ask? Did the professor understand what you wanted to ask? Was the professor’s answer in line with your question? And did the professor’s answer have any impact on your learning?

### Third stage

By means of the parallel transcription technique,^12^ the videos of the episodes were edited by adding the think-aloud interviews to have a differentiated point of view of the same S-P interaction, both from the expert professor and the students’ perspective. This new arrangement was called didactic sequence (DS) and was put together as follows: 1)the classroom context prior to the S-P interaction up to when the student asked the question; 2)the perspectives on the student’s question, recorded in the think-aloud interview, first the one from the professor and then the student’s; 3)the classroom context when the professor answers the question; and 4)the recollections in the think-aloud interview on the answer the professor gave in class, first the professor’s and then the student’s. For proper identification, the DSs were coded with the topic of the students’ question as follows: DS1: Valsalva maneuver; DS2: Refractoriness; DS3: Inotropism; DS4: Auricular valves; DS5: Sodium-potassium pump 1; DS6: lead D2 shows P wave; DS7: Internodal tracts; DS8: His bundle; DS9: Atrioventricular; DS10: Repolarization; DS11: ST segment; DS12: Sodium-potassium pump 2; DS13: PR interval; and DS14: TP wave. In the results, the following distinction between the voices of the participants was made: (DS#/P) for the professor and (DS#/S) for the students. 

Through the visualization of the didactic sequences, a first level of analysis was done through the identification of the connection between the professor’s meanings and those of the students. The 14 DSs were entirely transcribed by the researchers, as they required a careful selection and analysis to be organized in a matrix with the *Unit of Analysis S-P (i-e-a)-S´*, the elements of which are the following:^12^

Student (S): Type of student question.

Professor (P)

- Identification (i): How the professor perceives the student’s intervention for the identification of relevant aspects.
- Evaluation (e): Interpretation of the question for the identification of essential aspects for learning.
- Answer (a): Organization, elaboration, and identification of the type of answers.

Student (S´): Identification of the type of student reactions to the professor’s answer.

Afterwards, an analysis of the matrices was carried out by means of continuous comparisons in which similarities and differences in the data were contrasted to identify categories. This analysis was made during 2020 and 2021 in 20 monthly meetings of the research team from the public university of Medellín, and in 10 meetings with the principal investigator of the multicenter study. In these meetings the results were organized and refined. Likewise, the analysis was complemented in two socializations made in two international academic events organized within the framework of the multicenter study, one in Bogotá and the other in Medellín. 

This study was approved by the Research Ethics Committee of the Faculty of Nursing from the Universidad de Antioquia (Colombia). This nursing education institution granted endorsement for the study and the expert professor, with whom researchers had already been in contact, signed the informed consent. The professor also made it possible to get in contact with the two groups of students. For the recording of the classes, oral consent from the students was obtained, using also audiovisual recording, after presenting the objectives of the research. None of the participants refused to take part in the study. During the entirety of the research, willing participation was guaranteed, and efforts were made to maintain trust and respect for the participants. There were no conflicts of interest.

## Results

The expert professor who participated in the study is a 38-year-old male, who is a professional nurse, and who holds a graduate certificate in Basic Biomedical Sciences, a master’s degree in Critical Care and Emergencies and a master’s in Nursing. When the study was carried out, the professor had 11 years of professional experience as a nurse (during which he had some sporadic teaching experience) and 5 years as a full-time professor. The five interviewed students-four male and one female between 18 and 26 years old-were in the second academic semester of the Nursing Undergraduate Program. 

Four emerging categories and a meta-category were identified:

*First category. Identification of essential aspects: importance of prior knowledge*

The results show that, in order to link his meanings with those of the students during classroom interactions, the expert professor identified the essential aspects for learning through the students’ prior knowledge, since, as he stated on several occasions, each of the questions from the students *involves prior knowledge* (DS8/P), either from previous moments in the course *of what we have been working on during the semester* (DS10/P); or from their personal or family experiences, as *they [the students] have already seen it in their daily lives* (DS1/P); or from their work experiences when *they [the students] have seen it, have lived it, or have experienced it working as nursing assistants* (DS14/P). 

The search for this prior knowledge was recognized in the professor’s first recollection during the think-aloud interviews, when he stated that, when faced with the student’s question, *the first thing I thought about was… ‘Why is the student asking me this?’* (DS1/P) *or why did the student talk to me about pauses, if I haven’t even showed them the electrocardiogram?* (DS7/P). This unspoken concern opened an introspective path about the origin of the question, by comparing its elements with the main aspects of the topic in which it was asked, in order to understand that the student, by asking the question, brought up a concept, idea or topic, which, although it was similar or related to the subject under discussion, was new and different:

*Where did the student get the concept of hypertension? So much so that I have never talked about hypertension. Maybe the student got it from prior experience or from other courses* (DS3/P).

Therefore, the essential aspects on which the professor focused his attention were those new or differential concepts, sometimes confused or mistaken, represented in expressions, words or gestures, and their importance lies in the fact that they allowed him to identify the prior knowledge of the students in a given context: either the same class, another class from the same course, or the student’s personal, family or work experiences.

*Second category. Interpretation: connection between essential aspects and students’ mental processes*

The contextualization of prior knowledge facilitated *the interpretation* of these essential aspects to dive into the student’s mental process when stating the question. To this was added the knowledge the professor had of the students and their learning styles, because, as he said: *I have discovered that the students are very experiential* (DS13/P). Thus, the professor connected the essential aspects with the student’s mental processes to get to the experiential origin of the question, as can be seen in the following examples:

*Example 1: DS2-Refractoriness*

Student question: *During a heart attack is refractoriness very high or something like that?* (DS2/S). During the think-aloud interviews, the professor said that what he emphasized the most was the *interplay of words in common… As a professor, who has already had experience with them, who knows how they behave, how they talk, who knows some of their life experiences, what can I take? So, I give them an interplay of words from a theoretical perspective, which I illustrate with examples, and they give me back another interplay of words, which in some cases are common and in others are not. That is why they take me to doubting. That is why I need to stop to understand if what they are taking me back to is the concept* (DS2/P).

According to the professor, thinking of this *interplay of words* as the essential aspect led him to analyze quickly and immediately that the student was using two different words as synonyms, but that, *in essence, are the same*, not because they are similar words, but because he identified in them… *the ability to associate a learned concept and bring it here, even with a variant of the word, from ‘refractory period’ to ‘refractoriness’. Two different concepts, in different cells, but that he [the student] associated* (DS2/P).

According to the professor, identifying the correct association between *refractory period* and *refractoriness*, that is, the interplay of words used by the student, was possible thanks to the identification of the origin of the question, since this was one of the concepts that had been addressed in a prior moment of the course by the same professor: *I think what mattered the most there was the prior knowledge about ‘refractory period’, different from what I was explaining which was ‘refractoriness’, but they associated it properly […] that is, I had already talked to them about it and the already knew it* (DS2/P).

*Example 2: DS4- Auricular valves*

Student question: So, *do auricular valves close due to stimulus and ventricular valves due to pressure?* (DS4/S)

For the professor, the essential aspect of the question, the one that most attracted his attention, is the *so* in two ways: *what is initially more relevant to me is the emphasis he places on the question, and the second most relevant thing is the structure of the question* (DS4/P). About the first aspect, he emphasized *the strength with which he asked me,* in other words, *the strength of the question* (DS4/P); and about the second, the *so,* which according to him, represented a connection (continuity) between the professor’s prior explanation in class and what the student wanted to ask: *I have to interpret it as ‘Hey, if you are telling me this, so, does this have to be like this, or … can it be like this?’* (DS4/P). In other words, according to the professor, the *so* indicates an act of reflection on what he (the student) wants to ask, versus what the professor is teaching or saying (DS4/P). This analysis of the context of the classroom is what allowed him to arrive to the student’s mental process, [who] *makes an important association between what was learned before and what is now being learned. Before, we were discussing pressure; now I was not talking about pressure, but about blood flow and impulse; so, what he did was an association between the two* (DS4/P).

The interpretation of essential aspects through the recognition of the student’s mental processing was useful to the professor to confirm his understanding of the question and, above all, the student’s understanding of the topic.

*Third category. Organization of the answer: connection between prior knowledge and new knowledge*

After interpreting the student’s intervention, the professor organized the answer, starting from the essential aspects to *confirm* (DS2/P) and *ratify* (DS3/P) the connection between prior knowledge and new knowledge. This is how the construction of the answer began through dialogic processes in which the professor stimulated the student’s participation taking center stage. On the one hand, the professor identified that the student understood the topic *completely* (DS6/P), because, according to him: *That is the first thing I say: ‘This one understood what a vector of depolarization is’; internally I think about it* (DS6/P); and from this he asked counterquestions to motivate student’s participation in the construction of the answer, because *my excitement is reflected in returning the question to him, because he is basically giving me the elements of the answer* (DS6/P).

In this way, the professor broadened the expectation regarding the student and motivated him to complete his participation, internally anticipating the answer: *I kind of want him to tell me: ‘Because that’s how it depolarizes’. I’m thinking about the answer I want to hear, because I already elaborated it: ‘Man, the thing is, if you ask me such a brilliant question, you can answer it yourself: Why do you think is that?’* (DS6/P).

On the other hand, when the professor identified some confusion in the understanding of the topic because *the student explained the concept inadequately to me* (DS5/P), he constructed the answer based on the communicative intention of providing the necessary elements for the student to recover the prior knowledge, reminding him that *this does not work that way, we have already said it many times: ‘the sodium-potassium pump returns to homeostasis’* (DS5/P); and, furthermore, he says: *I have to go back and explain it again, so he [the student] himself would realize his mistake. That he himself would understand that there is a mistake* (DS5/P). To elaborate on the answers, on some occasions, he resorted to the memory of classmates to validate the understanding of prior knowledge by asking them: *Do you remember what the peak potential was in a normal cell? Not in this cardiac cell we are seeing, and they all say: ‘Oh, it’s that much!’. So, I assume they do know it. There is already a prior concept (*DS10/P).

Regarding the construction of the answer, it is important to note that one element that should be highlighted about the participant professor’s expertise was his ability to lead students to make a deep mental connection between their prior knowledge and the elements of new knowledge, since as he stated: […] *I can make a faster connection than the students. That is why I talk about the students who keep thinking for a while, as if they were still analyzing the concepts. And that is what happened in this case: being able to make that association, that interplay of words between prior knowledge and what is taught now. It seems to me that this is the point of meaning and what leads them to understanding* (DS2/P). For this reason, even if *the answer is made brief, short, concise, and to the point, I always try to explain and go further: to exemplify again* (DS3/P), in an attempt to reconnect the students with the origin of the question.

The answers of the professor, their organization, and the strategies to develop them indicate a system that connects prior knowledge with new student-centered knowledge. 

*Fourth category. Synchronization with the learning needs of students*

From the answers given by the professor, it is evident that there is an important synchronization between what the student wanted to ask and the professor’s answer, as the following testimony makes explicit: *he has that ability to answer to the point, where it is, without complicating and entangling things; and he answers your questions with assertiveness* (DS14/S). According to what the students said, the professor understood the question *one hundred percent, he got it very well and he knew how to answer it* (DS8/S). At the same time, they expressed that, in most cases, the professor’s answer was adjusted to their learning needs, not only because he assessed the understanding of the topic, since *he first told me that I was right about my input* (DS3/S); but also because he led the student to consolidate the relationship between prior knowledge and new concepts, because, as the student said: *he then made me associate the topics already learned with what we were learning at the time* (DS3/S). This coupling occurred even when the student appeared confused in the comprehension of the topic. In relation to this, one of them said: *The professor detected the small mistake, because he toys with you* (DS4/S), and also said that this is why the professor asked a counterquestion, which was even answered by the classmates: *I think that, in that moment, the classmates whispered: ‘No, due to pressure’* (DS4/S).

*Meta-category. Prior knowledge of the student: essential aspects for learning*

The cross-sectional analysis of the four categories showed that the identification of prior knowledge from the contextualization of the differential aspects of the student’s question, its interpretation starting from mental processes, and the organization of the student-centered answer, were essential devices for learning. Regarding the interaction of the expert professor during the class, the students said that they acquired, clarified, and ratified new knowledge because the professor *makes us reinforce the prior knowledge we have and allows an association of knowledge. That is, what we already know with what we are learning now* (DS3/S). According to what one student pointed out, this learning helped him *to grow as a nurse and to be prepared to take care of my patients in the best possible way* (DS8/S).

In addition, the professor verified the learning of students in two ways: the first was related to the conceptual clarity expressed by the students in the construction of their answers, since *I think it was very clear to them that they were two completely different phenomena; especially because I make the clarification between what would be trying to blow into a syringe to raise intrathoracic and intraabdominal pressures, as opposed to what is simply re-inhaling a gas in a bag* (DS1/P). The second refers to the verification of learning from nonverbal language, as in the case of a student who evidenced the acquisition of new knowledge with her *approving gesture, head nodding and confirmation that she understood* (DS9/P). 

## Discussion

The analysis of the PCK in this research has made it possible to find some essential aspects, among which the necessary exploration of prior knowledge stands out, with which a nursing expert professor established a route for connecting his meanings with those of the student, in favor of learning in a dialogic context. This ratified the assumption that has been proposed in education regarding the importance of starting from prior knowledge in order to achieve significant learning.^14^ The results show that the inquiries about prior knowledge made by the nursing expert professor were transversal during all interactions in the classroom and resulted from paying attention during the dialogues with the students to delve into their origin, and even to encourage their emergence in those cases in which they were not made explicit, with the purpose of building an answer articulated and contextualized with the discussed topic. With this, it can be inferred that the expert professor in this study used in his pedagogical practice what Ferreira *et al.* ^15^ call *cognitive mediation*, with which he optimizes learning opportunities in the classroom, consolidating the connection between the prior knowledge of the student and the new knowledge.

The exploration of this prior knowledge was possible in the practice of the nursing expert professor because it took place within the framework of dialogic processes, in which the students played an active role with constant interventions. These allowed the professor to identify, in the mental process of the students, the capacities to associate, relate, integrate, and connect this prior knowledge with the contents of the class, while at the same time they guided the pedagogical action, using the same mental processes of the students for the stimulation of reflective thinking and understanding of the topic.

This indicates that the nursing expert professor placed the analysis of the students’ conceptions in the complex constructivist dimension referred to by Martínez^16^ since he interpreted the prior ideas and mental processes of students as relevant constructions given by their interaction with the world, and used them for the students to reorganize their own system of ideas. The above complements what was expressed by Domínguez *et al.*^17^ in that, in order to achieve significant learning, it is not enough for the students to assume a committed and responsible role in their learning process, but it is necessary for the professor to propose strategies of active cognition to stimulate the processing, organization, and consolidation of knowledge; so that the teaching-learning process does not focus only on remembering content, but on leading students to reflect dynamically and to construct their own knowledge propositionally.

When characterizing PCK in experimental sciences professors (Biology, Chemistry and Physics) Agudelo *et al* ^7^ reported that each case of PCK is unique, because in employing the problem-solving method, each professor used unique structures according to their own disciplinary field, experience, and educational background for the interpretation of prior knowledge. In this regard, they reported that some of the participating professors privileged prior ideas related to disciplinary knowledge and thinking skills; others related them to mathematical operations; and still others to specific knowledge and to the nature of teaching^7^

Similarly, the PCK of the nursing expert professor is also a unique case. The difference with the results of Agudelo *et al*.^7^ is that the particular method of this professor was developed through a complex process whose central axis was the students’ experience, since he did not inquire about prior knowledge as abstract conceptions isolated from the subject, but placed them in the context in which they originated, such as the same class, another class or some personal, family, or work experiences of the students. That is, he articulated the prior knowledge of the students to their situated action^18^ because when he searched for the origin of prior knowledge he did not relate it only to disciplinary knowledge, cognitive skills, and specific knowledge, but contextualized it in the daily life of the students and thus built answers linked to their own experiences. 

The expert professor of this study started from the prior knowledge of the students during the classes and delved into the origin of that knowledge, to interpret it in the light of the students’ experiences and to articulate it to the knowledge he had about them, that is, their learning styles, interests, and needs. The knowledge of the students is a distinctive characteristic in the practice of the nursing expert professor and contrary to what is reported by other studies on novice professors. In this regard, Conceição *et al.*^5^ found that, lacking expertise, science education professors did not give importance to prior conceptions based on students’ knowledge and, therefore, did not include their background and previous experiences in their teaching strategies. In addition, the results revealed that the professor of this study got the students to articulate their prior knowledge with the new concepts, through the permanent interaction between the knowledge he had about the students and his expertise on the topic of cardiology. Both of these components were already recognized in the PCK as content knowledge and student knowledge,^6^ which in this research were observed through the professor’s permanent assessment of the students’ understanding of the topic, based on the types of questions most commonly asked by them and their gestures, detecting and correcting their mistakes during the class.

In this research, the interaction between content knowledge and student knowledge was also the teacher's ability to interpret learning difficulties based on the silence and some body language; in his ability to give a short or long answer, according to the characteristics and interest of each student; and in his skill to encourage each student to elaborate their own conceptual constructions, even when he had already anticipated it introspectively. When comparing the above results with those reported by Oztay *et al.*^6^ it can be deduced that both student knowledge and content knowledge are essential components of PCK, which ratify the expertise of the professor who participated in this study. Using a strategy similar to think-aloud interviews, called video-stimulated recall interviewing, Oztay *et al.*^6^ concluded that having knowledge on a topic does not guarantee using it. Thus, the chemistry professors who participated in their study, although having detected while teaching the students’ possible misconceptions and some of their difficulties in understanding the topic, reported that, due to their lack of teaching experience, they had encountered difficulties in recognizing and correcting them, specifically within the class. The difference with the nursing expert professor is that the chemistry professors did not inquire about the reasons underlying the students’ prior ideas, in order to deconstruct the misconceptions.^6^

The results presented here, in agreement with the PCK, also showed that the nursing expert professor uses appropriate pedagogical strategies to transform disciplinary or specific knowledge, in this case about cardiology, into knowledge that is understandable, relevant, contextualized and situated in the practice of the nursing student, which was manifested in the deployment of specific strategies for the development of the classes, since he frequently used metaphors, anecdotes and examples from daily life in a playful manner in the elaboration of the answers. These strategies, according to the analysis, were useful to motivate students, increase their participation and improve their understanding of the concepts by placing these strategies in contexts that were familiar to them. Previous studies on PCK reached similar conclusions. Some reported that philosophy professors frequently resorted to everyday examples to capture students’ attention.^2^ Others found that chemistry professors tried to connect scientific knowledge with the students’ daily lives, eliciting classroom discussions, presentations, animations and analogies to keep them active.^6^ Almonacid *et al.*^8^ identified that physical education professors tried to motivate students through playful strategies and participatory, individualizing, creative, and socializing teaching styles, convinced that motivation during classes leads to quality learning.

This ability of the professor to transform his disciplinary knowledge about cardiology into a knowledge apprehensible by the students was also demonstrated by the use of dialogic strategies to stimulate the student to elaborate the appropriate answer, showing them that they can believe in their abilities. This was evident when he used questions and counterquestions to validate learning, leading the student to verify the deductions and associations that he himself made. Similar results were shown by Cruz *et al.*,^2^ when they stated that the purpose of permanent dialogues and relevant questions was to develop dialogic, critical, and interpretative skills.

The expert professor also used dialogic strategies when he identified confusion or mistakes in the students’ questions and invited them to make contrastive and validation exercises between their prior knowledge and the new knowledge to construct adequate conceptualizations. This is similar to what was found by Timo,^19^ who reports that, before exercises with erroneous results, the student looks for the faults in their mental schemes, in a comparative exercise of their correct or incorrect approximations that, consequently, forces them to their mental schemes revise again according to the correct solutions.

Thus, it is valuable in this study that even for the expert professor, the students’ mistakes were assumed to be opportunities for learning in an environment of trust. In this regard, Palominos *et al.*^20^ showed that the naturalization of errors is an aspect that enables learning. Therefore, their acceptance is not only a way to advance in the acquisition of new knowledge, but also helps to minimize negative emotions towards mistakes, achieving greater comfort and security in the learning process.

Due to all of the above, the practice of the expert professor of this study acquired a profound pedagogical meaning, since he used innovative and relevant didactic strategies in favor of learning, which is explained by the findings of Jaramillo,^21^ in the analysis of modern pedagogical tendencies. This author affirms that the students do not learn a copy of what they observe around them, but that the professor’s mediation with innovative, creative and integrating strategies helps them to reelaborate their own knowledge. Consequently, the expert professor’s ability to recognize the student as a subject protagonist of their own learning is exposed, that is, he uses a pedagogical practice centered on them and crossed by their knowledge, in the context of negotiated interactions, for an authentic learning that, attached to their mental structures, is built from their social reality and their interaction with the world.^22^

Conclusion. The prior knowledge of the students is the essential aspect identified, interpreted, and organized by the expert professor to achieve significant learning in them. Prior knowledge is, therefore, the device with which the professor articulates two characteristics of PCK, namely, content knowledge, that is, the topic of the class, the subject matter, and student knowledge, in order to achieve a higher level of thinking. As described above, when the professor considers the prior knowledge of the student, explores it, organizes it to recognize the students’ level of understanding and, through didactic strategies, allows them to connect it with new knowledge, a clear teaching posture centered on the student and concerned with the student’s learning is evident. Such learning corresponds to an educational paradigm that places the student at the center of the educational process, recognizing each student as a unique being, with his or her capabilities, needs, knowledge, interests, experiences, as well as learning styles and paces achieving that learning. 

Furthermore, prior knowledge is an essential element that sets the path for an experiential pedagogical practice that goes beyond theoretical and abstract concepts. The expert professor shows the ability to transform the students’ prior cognitive knowledge into experiential knowledge, as he focuses his action on the mental processes and real experiences of the students, since he places them in the context in which they originate, in order to understand them and build an answer that adapts to their learning needs. In this case, their experience plays a fundamental educational role during the pedagogical interaction in the classroom, by connecting the prior knowledge of the students with their disciplinary knowledge.
